# Michigan Dental Association

**Published:** 1866-03

**Authors:** 


### MICHIGAN DENTAL ASSOCIATION.

This society met in Detroit on Tuesday the 30th of January. Quite a large number of the profession in the State was in attendance. All exhibited a lively interest in the society, each taking part in the proceedings. There where a number of good papers read, some of which with the proceedings will be found in this number of the Register.

The propriety and advantages of securing an act of incorporation was discussed, and it was finaly resolved to become an incorporated body. A committee was appointed for that purpose, with instructions to proceed at once to its accomplishment.

We think it would be quite advantageous and appropriate for all of our more important professional societies to avail themselves of the privileges accruing to incorporated bodies.

The establishment of a dental college in the state of Michigan, is receiving a large share of attention, and consideration by the profession of that state, and we doubt not that something worthy of their noble effort, will ere long be accomplished.

This society consists of true and good men, who will carry forward to a successful result, every enterprise in which they may engage.

